# Building an Interactive Geospatial Visualization Application for National Health Care–Associated Infection Surveillance: Development Study

**DOI:** 10.2196/23528

**Published:** 2021-07-30

**Authors:** Shuai Zheng, Jonathan R Edwards, Margaret A Dudeck, Prachi R Patel, Lauren Wattenmaker, Muzna Mirza, Sheri Chernetsky Tejedor, Kent Lemoine, Andrea L Benin, Daniel A Pollock

**Affiliations:** 1 Division of Healthcare Quality Promotion Centers for Disease Control and Prevention Atlanta, GA United States; 2 Department of Medicine Division of Hospital Medicine Emory University School of Medicine Atlanta, GA United States

**Keywords:** data visualization, geospatial information system, health care–associated infection

## Abstract

**Background:**

The Centers for Disease Control and Prevention’s (CDC’s) National Healthcare Safety Network (NHSN) is the most widely used health care–associated infection (HAI) and antimicrobial use and resistance surveillance program in the United States. Over 37,000 health care facilities participate in the program and submit a large volume of surveillance data. These data are used by the facilities themselves, the CDC, and other agencies and organizations for a variety of purposes, including infection prevention, antimicrobial stewardship, and clinical quality measurement. Among the summary metrics made available by the NHSN are standardized infection ratios, which are used to identify HAI prevention needs and measure progress at the national, regional, state, and local levels.

**Objective:**

To extend the use of geospatial methods and tools to NHSN data, and in turn to promote and inspire new uses of the rendered data for analysis and prevention purposes, we developed a web-enabled system that enables integrated visualization of HAI metrics and supporting data.

**Methods:**

We leveraged geocoding and visualization technologies that are readily available and in current use to develop a web-enabled system designed to support visualization and interpretation of data submitted to the NHSN from geographically dispersed sites. The server–client model–based system enables users to access the application via a web browser.

**Results:**

We integrated multiple data sets into a single-page dashboard designed to enable users to navigate across different HAI event types, choose specific health care facility or geographic locations for data displays, and scale across time units within identified periods. We launched the system for internal CDC use in January 2019.

**Conclusions:**

CDC NHSN statisticians, data analysts, and subject matter experts identified opportunities to extend the use of geospatial methods and tools to NHSN data and provided the impetus to develop NHSNViz. The development effort proceeded iteratively, with the developer adding or enhancing functionality and including additional data sets in a series of prototype versions, each of which incorporated user feedback. The initial production version of NHSNViz provides a new geospatial analytic resource built in accordance with CDC user requirements and extensible to additional users and uses in subsequent versions.

## Introduction

The National Healthcare Safety Network (NHSN) is a web-based system developed and maintained by the Centers for Disease Control and Prevention (CDC), and used by health care facilities, the CDC, and other agencies and organizations for surveillance of health care–associated infections (HAIs), antimicrobial use and resistance, and other health care events and processes. NHSN data serve a variety of purposes that include infection prevention, antimicrobial stewardship, and clinical quality measurement. The CDC launched the system in 2005 and its current surveillance coverage extends to over 37,000 health care facilities nationwide. Because of state and federal HAI reporting requirements, NHSN’s geographic and health care facility coverage is extensive and the granular HAI data and summary statistics available for use are voluminous.

Health care facilities submit data to the system in accordance with NHSN’s surveillance protocols, and the facilities access the system’s analytic features to produce summary statistics for their own facility, including the cumulative attributable difference (CAD) [1]—a benchmark of the difference between observed and predicted HAIs—and the standardized infection ratio (SIR) [2]—a measure of the ratio of observed to predicted HAIs. Summary statistics enable intrafacility and interfacility comparisons for individual health care facilities and groups of facilities, and also provide a means to assess HAI prevention needs or progress at local, state, and national levels.

All data that health care facilities submit to NHSN are facility identifiable and can be linked to a street address, which enables analysis and display of NHSN data by the geographic location of each reporting facility. Most of the data that hospitals report also specify the patient care location within the hospital where the data originated (eg, intensive care unit [ICU], and, more specifically, by type of unit, such as surgical or medical ICU). For analytic purposes, NHSN data may be aggregated at a variety of geographic levels, from patient care locations within individual facilities to facility, regional, state, and national levels. NHSN analyses also may call for data to be aggregated according to predetermined time intervals, such as calendar quarter and year, or by customized data ranges.

The wide variety of granular data submitted to NHSN, the increasing volume of NHSN data and complementary data available from external sources, and the broad range of NHSN data analyses that are produced, planned, or could be made possible present an opportunity to leverage the large amount of data on a “big data” platform for NHSN data users. Among the basic requirements are systematically organizing the massive amount of data available because of NHSN’s surveillance coverage and making those data readily available to NHSN data analysts in ways that help maximize the usability and usefulness of the data for analytic purposes.

The importance of data visualization for exploratory data analysis is axiomatic across many disciplines. In epidemiology, visualization of geospatial data can reveal meaningful associations between a local site, such as a health care facility, and its surrounding environment [3]. Compared with traditional data representations, such as tables or static figures, and particularly when the data can be characterized as large volume, high complexity, and highly disparate [4], data visualization based on a geographic information system (GIS) display offers enhanced opportunities to use them for public health purposes, such as for infectious diseases prevention and control. In practical terms, data visualization bolsters public health efforts, including outbreak detection and response activities [5,6].

At the CDC and throughout public health, data visualization is an important means of enhancing efficiencies and scalability of data analysis and dissemination [7]. Data visualization tools and platforms developed at the CDC’s enterprise and division levels typically serve domain-specific programmatic needs, such as environmental and behavioral health. These programmatic systems tend to focus on data presentations at geospatial levels—municipal, county, state, and national—located above the health care facility level. The paramount importance of facility-level data for HAI surveillance and prevention places a premium on aggregating, processing, and exhibiting data at a more granular level than is typical for other public health domains, while also enabling HAI data to be visualized at higher geospatial levels. Commercially available, ready-to-use visualization tools provide an array of important capabilities, such as rendering simple visual displays of bivariate analytic results [8]. However, the shortcomings of these systems typically include limited capacity to represent analytic results that involve multiple variables, aspects of data in parallel, or complex relationships between various data sources [9]. These limitations led the NHSN program to make the decision to develop a system for visualization of NHSN data sets—NHSNViz. Among the most important requirements identified and prioritized for the NHSN platform are the capacity to capture the logic embedded in data and to stimulate knowledge discovery. Therefore, consolidating multiple data sources and layers into the same space is a prerequisite. Additionally, the underlying connection between multiple variables of data needs to be explicitly expressed via proper user interaction design, which can streamline otherwise complicated surveillance inquiry tasks. All these requirements would be better satisfied with an ad hoc solution which is not limited by predefined visualization templates.

In summary, this project is driven by the following research questions: in terms of HAI prevention analyses, “What are the critical data sources and elements,” “What is the most intuitive and user-friendly visualization representation of each variable,” and “How could multiple facets of the various data sets be integrated into the same display systematically?”. By leveraging the expertise of infection preventionists, these topics have been explored in depth. In the following sections, we introduce our preliminary responses for these research questions as well as the methodology of our analyses process.

## Methods

### Overview of Development of NHSNViz

**Figure 1:**
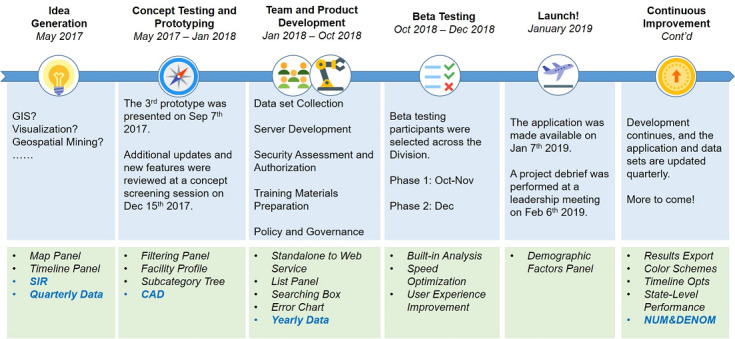
NHSNViz development timeline. CAD: cumulative attributable difference, GIS: geographic information system, NUM&DENOM: numerator and denominator, SIR: standardized infection ratio.

Statisticians, epidemiologists, data scientists, and other stakeholders, including policy and communications staff, spurred NHSNViz development (Figure 1). Their input was translated into technical requirements for the system’s design and functionality, which in turn were applied in an agile software engineering life cycle characterized by a short feedback loop and sequential improvements that incorporated stakeholder feedback [10]. In particular, all requirements proposed by stakeholders are decomposed, itemized, and documented systematically. Each feature is then prioritized based on its value and the overall business strategy. Finally, the design and implementation are reconfirmed via sketch, paper interface, or prototype to guarantee transparent communication between stakeholders and developers.

The project began in earnest in mid-2017 following an increase in stakeholder requests for a ready-to-use set of tools that would facilitate geospatial analysis and visualization of NHSN data. These requests prompted an initial round of brainstorming conducted by nominal group techniques, formal requirements gathering based on group interviews, rapid prototype development followed by a user observation test, approval for a proof-of-concept project, organization of a development team, and initiation of a project aimed at producing a beta test version. In October 2018, the beta test version was released and tested by over 20 individuals, drawn from diverse disciplinary backgrounds and a variety of programmatic units within CDC’s Division of Healthcare Quality Promotion. All beta testers completed training with a 1-hour online learning course. Most users easily comprehended the operations of the system. Afterward, user feedback was collected via either a survey form or a structure interview. These suggestions are primarily related to the choice of visualization representation, for example, how to represent “null” value on the map and how to highlight an increase or decrease on the timeline.

Beta test user experience and recommendations guided further development of the NHSNViz application, which was launched for initial production use in January 2019. Since then, user input has led to additional NHSNViz enhancements, specifically the addition of new analysis and visualization features and new NHSN data sets.

NHSNViz’s continuous development and release cycle provides opportunities for incremental improvements while retaining a core set of maintenance processes and system features:

- Spatial locations of individual health care facilities are gathered via geocoding, providing an aid to identifying facilities and geographic areas where HAI prevention progress has been made or has yet to be achieved.
- Hospital characteristics, including hospital type (eg, major teaching hospital), number of hospital beds, and hospital network affiliation, enabling rapid assessments of individual hospitals with high or low SIRs.
- Quarterly and yearly aggregations of granular HAI data and summary statistics, such as numerator and denominator data, SIRs, and 95% confidence intervals are displayed. The availability of SIRs for each facility and for individual patient care locations/units within facilities promotes in-depth understanding of where HAI prevention resources are most needed.
- Demographic data acquired from external sources on income and census statistics provide additional data layers to support policy- and population-related analysis.

All these requirements eventually spawned a high-dimensional, high-complexity schema. To build such a big data application, modeling the relation among multiple data elements and constricting response time are the 2 major technical challenges to be addressed.

### Geocoding

Geocoding, which transforms physical locations such as mailing addresses to latitude- and longitude-based geographic coordinates, is an essential component for GIS applications. We used the Geocoding web service of Google [11] to obtain coordinates information for the NHSNViz project. As a part of the data collection process, the health care facility address that facilities report to NHSN in NHSN’s annual survey, including street, city, and state and zip code, was used to invoke the Geocoding service, and then the coordinates were parsed out from the JSON format response.

### Data Format and Preparation

Indexing and merging multidimensional tabular data into a single entity helps to optimize querying performance. The JSON format provides a light-weight semistructured norm for hosting multidimensional data sources. The particular JSON format specification, GeoJSON, is designed for encoding geographic data, with flexible customization of attributes as needed. We used the Point geometry type of GeoJSON to represent health care facilities, which enables transformation of each facility’s profile and measurement to subattributes under its “property” field. Coordinates collected from the geocoding process are inserted into the “geometry” field. This data transformation is completed offline as a part of a prior processing step to optimize the client-side performance.

### Interactive Map

An interactive map serves as the background canvas for a GIS system. Leaflet is a prevalent open-source JavaScript library for visualizing interactive maps. It is lightweight and yet provides the most commonly used map features such as layers, markers, and choropleth maps. Leaflet supports GeoJSON format directly. We apply this framework to NHSNViz by converting each JSON file entry (ie, a health care facility) to a “dot” marker on the map. The color of each “dot” and the corresponding legend are used to depict the level of a value, such as SIR or CAD.

### Visualization

The graphical form that users want to see when they visualize data depends on specific analytic interests (eg, basic frequency distributions, temporal trends, geographic comparisons, or other data relationships). AnyChart is a JavaScript library for rendering charts that takes into account a wide range of user needs and preferences. It supports more than 70 flexible and interactive chart types. We leveraged this versatility, in conjunction with an analysis of NHSN data users’ requirements, to enable NHSNViz users to render a variety of charts:

- Stacked column chart: This is used to represent data with multiple segments. It demonstrates how each segment contributes to the overall number and displays the changes overtime.
- Mosaic chart: Also known as the heatmap chart. It represents a data matrix while each cell uses different colors to represent different values.
- Waterfall chart versus Column chart: Both represent time-based data series. However, waterfall chart highlights the change between data points using floating columns while the column chart represents absolute value with the length of columns. With markers showing the upper and lower bounds, a column chart works as an error chart and could be used to display variability details such as the confidence interval of SIR.
- Treemap chart: This renders categorical data in a hierarchical way. For comparison purpose, it displays the proportion of every subcomponent and exhibits the value of each simultaneously, in other words, 2 facets of the entities could be visualized in parallel.

Use of these chart types in NHSNViz is illustrated in the “User Interface” section.

## Results

### System Architecture

NHSNViz’s underlying architecture is a classic client–server design with an HTML/JavaScript-based front end. When the server is started, all data and configuration files are loaded from a Windows shared drive with a JCIFS SMB connection. This design isolates the relatively stable source code from the regularly updated configuration parameters and data sets. To optimize NHSNViz’s client-side runtime performance, all required data are downloaded in batch when the application is opened in a web browser. Next, all querying and filtering interactions with the application are completed on the client side, which minimizes the hardware and bandwidth requirements for the server. Technical specification of the server are as follows:

- OS System: Linux CentOS 7
- Server: Tomcat 9
- JavaScript Libraries: Leaflet 1.5.1, AnyChart 7.4, DHTMLX 5.1

### User Interface

**Figure 2:**
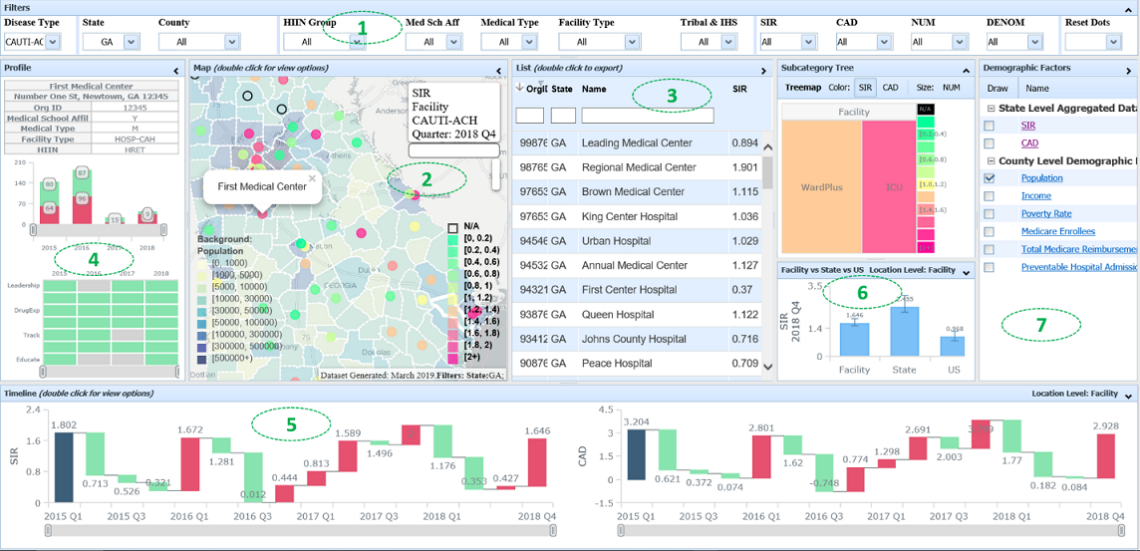
NHSNViz System Interface (Synthetic Data).

The resulting user interface can represent complex analytic results and consolidate multiple variables as well as multifaceted relationships between them. NHSNViz’s JavaScript-based interface leverages state-of-the-art visualization libraries, including Leaflet, AnyChart, and DHTMLX, which enables integration of NHSN data sets and the supplementary demographic data sets into a single-page dashboard (Figure 2).

This 1-dashboard, multiple-view design enables both experienced users and beginners to navigate easily across different geographic locations and periods. Users can conveniently browse demographic characteristics for selected geographic areas, clinical quality performance measures previously calculated and made available through NHSNViz at the health care facility, state, and national levels, and health care facility attributes such as bed size and medical school affiliation. These interactive features enable dynamic queries based on user’s operations.

A user may choose panels of interest to customize the layout of the interface for a specific task, and then navigate through different HAI types, time points, locations, and facility aspects.

To configure the Filters Panel (1), the user can focus on facilities displayed via Map (2) and List (3). Selecting either a dot on the map or an entry on the list will render detailed profile information and performance measures of the facility. One can also export the data set displayed on the List directly into a CSV file.

The Profile Panel (4) displays hospital bed sizes, including both ICU and non-ICU locations, using a stacked column chart, enabling data visualization for each subcategory segment. Additionally, the status of hospitals’ antimicrobial stewardship programs is represented by a mosaic chart, which is usually used to illustrate a categorical data matrix.

The Timeline Panel (5) at the bottom of the NHSNViz display enables access to clinical quality measures for calendar quarters or years, presented via a column chart or waterfall chart, highlighting the absolute or relative values, respectively. The Right Panel (6) displays additional performance details such as the Treemap chart representing patient care location groups within a facility that has a tree structure hierarchy and allows drill-down; the color of each unit represents a performance measure such as SIR while the size represents either the denominator or numerator value. In addition, both the Timeline and Treemap are interactive. The user may update performance measures displayed on the map and list to certain time or clinical unit by simply clicking on the corresponding data point.

The Demographic Factors Panel (7) supports aggregated measures and external complementary data sets. These data could be rendered as a Choropleth map to provide a resource for advanced data analysis.

According to the feedback collected during the beta test, we have polished the application further to optimize the user experience. The enhancements include but are not limited to adding different color themes to ease the interpretation of data, enabling direct data and image export through the interface, as well as providing extra tooltips and messages to smooth the learning curve. All these updates have been reviewed by the proposers and well received.

### Example Use Cases

**Figure 3:**
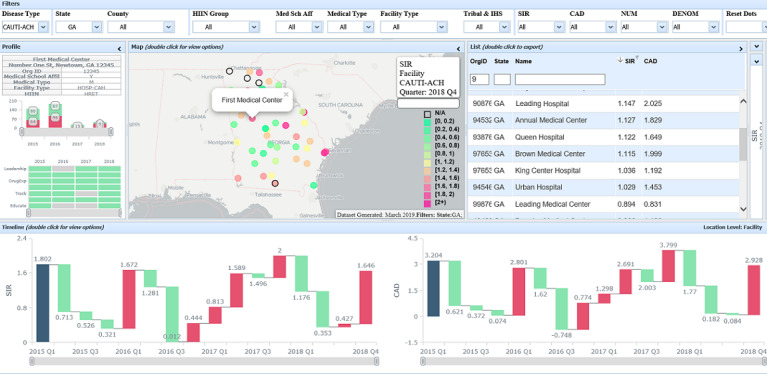
Example Use Cases (Synthetic Data).

A typical NHSNViz use case is identification and prioritization of health care facilities in a state health department’s jurisdiction for purposes of initiating or intensifying targeted HAI prevention efforts (Figure 3). First, a user can select a single state and specify a range of health care facility clinical performance scores from the Filters panel, which yields a subsetted list of the facilities that meet that criteria for targeted prevention. Next, the user can open the List panel, sort facilities in descending order by SIR or CAD, and inspect them individually. In addition, the user may browse the filtered results using the map, probing the distribution of high incidence or regions of high concentration.

An additional use of NHSNViz is reviewing the performance score distribution of a facility over a specific period. After choosing a facility via either the Map or the List, the user may configure view options of the Timeline panel to browse SIR and CAD measures by quarter-year or year. To complement this comparison, a user can view state- and national-level performance over the same time units and period. Lastly, to enrich the context for data analysis, demographic information on the surrounding area, such as population, income, and nearby airports, could be rendered on the map as well.

## Discussion

NHSNViz achieved its primary goals of making NHSN data more readily accessible for users, enabling easy data visualization across an array of user-selected, graphical displays, and providing a means for users to gain new insights from data displayed in temporal and geographical forms. In particular, the application facilitates access to an extensive amount of high-dimensional data across multiple complex metrics which can be used for analysis and action at all geographical levels of public health. In this study, we have explored a large amount of data sources related to HAI prevention and selected the most useful ones. For each data set, we picked the proper visualization method to exhibit the pattern or highlight the trend, facilitating user’s interpretation of data. In addition, all the facets of the various data sets have been indexed in a systematic way based on the geographic and temporal information, which allows users to navigate through different facilities and periods conveniently. These experiences could serve as a reference for other visualization applications and systems addressing the same topic. HAI prevention is a joint effort made by the facilities, regions, and nation. Visualizing HAI data expedites data analyses and benchmarks the prevention advancement, therefore drives infectious preventionists toward conquering HAI.

Launched in January 2019, NHSNViz serves as a key resource for CDC data users in assessing HAI prevention and a platform for further enhancements and extension of the system’s features to additional external users. By the end of 2019, the users of NHSNViz had invoked about 20,000 map tile requests in total. The 2-year development and maintenance experience also established guidelines for future projects. An external facing version of the application has been discussed and is in the planning stages, so the data could be shared with NHSN user groups with access control. Although adding supplementary data sets on the fly is not yet supported, additional requests for application features and data sets are addressed carefully in an agile way to enable continuous improvement. Currently the NHSN data sets used by the application are updated on a quarterly basis. Formal quality assurance process is performed by a combination of validation script and manual review. During each data preparation process, the raw data sets are scanned by a program to ensure the completeness and consistency of data. Whenever the application is populated with an updated data set, the quality assurance team will manually review and test the rendering of data to guarantee that the new data elements are properly represented.

Careful evaluation of NHSN’s different data types, in terms of their specific characteristics and their uses for various surveillance and prevention purposes, was a key consideration and a vitally important design determinant throughout the NHSNViz development process. This user-centered approach governed decisions about which graphical displays and interface features would help reveal data in the most meaningful way and optimize the application’s value [12].

A second overarching influence on the application’s design was the goal of enabling NHSNViz to serve as a resource to inspire knowledge discovery, hypothesis generation, analytic exploration, and data presentations. The 1-page dashboard design helps users to view and explore different data sources simultaneously in an integrated way [13]. Promoting ease of learning and inspiring user insights were top priorities and are reflected in the intricately engineered yet easy to operate system interface.

Further, we have identified several additional directions for future enhancements. GIS information resources can activate access to meaningful background data, prompt linkages between the health care facility’s geographic location and contextual social and economic factors, and support advanced analysis such as cooccurrence and network [3]. Enriching built-in geospatial mining functionalities could significantly automate and reinforce data analysis. For example, a thematic map, which is also known as a heatmap, could be created based on density and severity of data points. Another example of this direction is the analyses related to COVID-19. Rendering the distribution of COVID-19 cases as the background information may support the assessment of pandemic influences on HAI prevention needs or efforts. Such an artificial layer could expose geographic clustering of unexpected instances, which may be adopted to target problem areas of HAI. Another potential enhancement for analytics is to enable outbreak detection, such as the timely hotspot analyses for COVID-19. This feature requires near real-time data feed, and then spatial–temporal information would be assessed collectively using diagnostic algorithms [14]. Such a predictive early warning model may portray threats even before human’s cognition, which promotes timely information dissemination for epidemiology surveillance.

In summary, as a ready-to-use application, NHSNViz enables NHSN data users to gain access to a wide array of surveillance data with complex relationships beyond what is typically available for purposes of analysis, visualization, and reporting.

## Abbreviations

CAD: cumulative attributable difference
CDC: Centers for Disease Control and Prevention
GIS: geographic information system
HAI: health care–associated infection
ICU: intensive care unit
NHSN: National Healthcare Safety Network
SIR: standardized infection ratio
